# Asymptomatic *Plasmodium* Infection and Associated Factors in Selected Districts of the Kaffa Zone, Southwest Ethiopia: A Cross-Sectional Study

**DOI:** 10.1155/2023/4144834

**Published:** 2023-04-13

**Authors:** Tadesse Duguma, Eyob Tekalign, Mitiku Abera

**Affiliations:** Department of Medical Laboratory Science, College of Health Science and Medicine, Mizan- Tepi University, Mizan-Aman, Ethiopia

## Abstract

**Background:**

Malaria remains a serious public health problem, particularly in resource scarce areas of the world. The number of malaria cases has dropped remarkably in Ethiopia over the last decade, and efforts to eliminate the disease are underway. Asymptomatic infections may pose significant challenges to the elimination program. The essence of this study was to assess the prevalence of asymptomatic *Plasmodium* infection and the associated factors among communities of the selected districts in the Kaffa zone.

**Materials and Methods:**

April to May and September to October 2021, were the two seasons in which the community-based cross-sectional survey was conducted. Capillary blood from a finger prick was examined by light microscopy (LM) and screened using rapid diagnostic tests (RDTs). The participants' sociodemographic characteristics and malaria prevention measures were collected using a pretested semistructured questionnaire. Data entry and analyses were carried out using EpiData and SPSS version 25.0. Logistic regression (bivariate and multivariable) analyses were carried out to assess the possible associations between the dependent variable and the associated factors.

**Results:**

566 study participants were involved in the two cross-sectional surveys, including 234 male and 332 female subjects with a mean age of 18.486 (SD ± 15.167). Thirty-eight blood samples (6.7%) were found to be positive for *Plasmodium* species tested by both LM and RDT. Last night's use of long-lasting insecticidal net (LLIN) (AOR = 2.448, 95% CI: 1.009 5.938, *p*=0.048), presence of eave (AOR = 4.144, 95% CI: 1.049–16.363, *p*=0.043), and house sprayed in the last year (AOR = 5.206, 95% CI: 2.176–12.455, *p* < 0.001) were among factors that showed significant association with asymptomatic *Plasmodium* infection.

**Conclusion:**

The asymptomatic *Plasmodium* infection prevalence recorded in the study area was low. Last night's LLIN usage, the presence of an eave, a house sprayed in the last year, and the presence of stagnant water near the home of the study participants were among the factors associated with an increased risk of catching the disease.

## 1. Introduction

### 1.1. Background

Many countries in the world are still suffering from malaria, the deadliest and most common protozoan infection in humans. 627,000 malaria deaths were reported in 2021, which is also evidenced by an increase of the cases from the previous two years, 2019 and 2020, which accounts for approximately 95% of cases, with WHO African region countries accounting for most of this increase [1, 2]. Almost half of the world's nations are now malaria-free as a result of integrated malaria-fighting efforts over the last decade and a half [3].

Malaria transmission in Ethiopia has a dynamic pattern, with unstable and seasonal transmission occurring in the majority of the country's transmission areas. Perennial transmission occurs in the country's western lowlands, where the climate is conducive to malaria transmission all year [4]. Altitude and rainfall are two environmental and climatic factors that have a major impact on transmission. The two most prevalent forms of human malaria in Ethiopia are *P. falciparum* and *P. vivax* [5]. In high transmission areas, continuous exposure to *Plasmodium* parasites leads to partial immunity and, consequently, creates asymptomatic carriers [6]. When transmission conditions are unstable, which is usually due to seasonal factors, asymptomatic malaria can be seen to a lesser extent. Asymptomatic infections are a reservoir for parasites and a source of new infections when mosquito populations are low as they may carry the gametocyte stage of the parasite [7–9]. The frequency of asymptomatic parasitemia is influenced by the area's endemicity for malaria, the length of time spent there, age, the development of partial immunity from prior exposures to the disease, gender, use of bed nets, and genetic background [8]. Asymptomatic malaria can be used as an indicator for assessing the effectiveness of malaria vector control programs, including their implementation and ongoing use [8]. Light microscopy is recommended by WHO as the gold standard for detecting symptomatic malaria, but its performance in detecting asymptomatic infections, particularly in low-endemic settings, is generally poor [10]. Individuals with an axillary temperature of 37.5°C whose blood sample reveal malaria parasite are considered asymptomatic carriers along with the absence of malaria-related symptoms [11].

As vector control interventions, including IRS and LLINs, have been widely used in the last decade for the control of malaria, the burden of malaria has significantly declined in many parts of Ethiopia. These vector control interventions have likely played a vital role in the control of malaria [12, 13]. Drug resistance in asymptomatic individuals could further complicate the elimination efforts as they may spread the drug-resistant genes [14]. The malaria control strategies, which are typically dependent on the passive detection of cases in health facilities and treating them, frequently do not address asymptomatic infections. Active case detection, which identifies asymptomatic instances, is costly and has not been used frequently by Ethiopian medical facilities up to this point. The majority of people in malaria-endemic areas, however, do not exhibit any symptoms [14, 15].

Asymptomatic *Plasmodium* infection is impeding Ethiopia's efforts to eradicate malaria by 2030. As a result, it is crucial to ascertain the disease prevalence in the study area. That could aid in lowering the local parasite reservoir and in developing methods for effective control and, eventually, elimination.

## 2. Materials and Methods

### 2.1. Study Area and Period

This study was conducted from April to May and September to October 2021 in selected districts of the Kaffa zone (Decha and Gimbo), which are among the 13 districts in the Kaffa zone. These districts were known for their history of having high malaria transmission. The research was carried out 451 kilometers from Addis Ababa, the country's capital city. According to the CSA's 2007 Census, these districts have a total population of 128,887, of which 64,438 are men and 64,449 are women (Decha), and 89,892, of which 44,774 are men and 45,118 are women (Gimbo).

### 2.2. Study Design

A community-based cross-sectional study was employed.

### 2.3. Source Population

All residents in the selected districts (have a previous history of high malaria transmission) of the Kaffa zone.

### 2.4. Study Population

All individuals within the selected households who took part in the study.

### 2.5. Inclusion Criteria

All residents aged 6 months and older, who did not present any malaria-related symptoms/Apparently healthy individuals (with no complaints related to malaria symptoms) were taken as eligible to participate in the study.

### 2.6. Exclusion Criteria

Participants who were not able to respond to the questionnaire and were unable to provide a blood sample for different reasons. Individuals who did not consent to participation, as well as those who were taking antimalarial medication two weeks before data collection, were excluded.

### 2.7. Sample Size Determination

The sample size was calculated using the formula for a single population proportion.

(1) $$\begin{array}{c} n=\frac{\left({\left(Z\alpha /2\right)}^{2}\;p\left(1-P\right)\right)}{d^{2}},\\ =\frac{\left({}^{2}0.067\left(1-0.067\right)\right)}{{⟦0.03⟧}^{2}},\\ =266.8\approx 267\text{.} \end{array}$$

 *n* = the minimum sample size required for the study. Z*α*/2 = is the standard normal variable at a 95% confidence level (1.96).*p* = proportion of the population that has asymptomatic malaria infection was taken from a study in Northwest Ethiopia (6.7%) [16] *d* = margin of error (0.03).

Adding a 10% nonresponse rate, the final sample size was 293.7 ≈ 294.

In the two-round surveys, the total sample size was taken to be **588**.

### 2.8. Sampling Technique

Of the two selected districts, Decha with 43 villages and Gimbo with 35 villages, six villages, namely (Shellew, Bulkabul, Awurada, and Ketema) villages from Decha district and (Kecho, Hiberet, and Ufa) villages from Gimbo district, were selected purposely due to their previous history of being severely affected by malaria. The sampling frame of households was obtained from the health posts of the selected villages. To recruit the desired sample size, a systematic random sampling method was used with an interval of “*K*” of 3, but the first household was selected randomly. Finally, all individuals in a household that was available during the survey and fulfilled the inclusion criteria were included. After obtaining written informed consent from parents/guardians, all members of the randomly selected households were asked to give a blood sample.

### 2.9. Data Collection Tool and Method

#### 2.9.1. Questionnaire Data

Sociodemographic information and data on risk factors for malaria were collected using a pretested semistructured questionnaire. The questionnaire contained information about the study participants' sociodemographic characteristics (age and gender), housing conditions, indoor residual spray, the presence of stagnant water near the study participants' homes, bed net usage, and bed net availability for sharing the living house with domestic animals. The questionnaire was first prepared in English and then translated into the local language, which is spoken by the residents of the villages or “kebeles.” The data were collected by laboratory professionals and health extension workers.

#### 2.9.2. Blood Sample Collection and Processing

Finger prick samples were collected from consenting individuals for blood film preparation for microscopic examination and multispecies malaria rapid diagnostic tests. Five microliters (5 *μ*l) of blood sample and 2 drops (60 *μ*l) of buffer solution were used for the RDT malaria screening following the manufacturer's instruction, whereas, 6 *μ*l and 2 *μ*l of blood sample were taken for thick and thin blood film preparation.

#### 2.9.3. Pregnancy Screening

A minimum of 1 milliliter of urine sample was collected from female study participants who responded “yes” to the questionnaire “are you pregnant?” and tested using human chorionic gonadotrophin (HCG) kit to confirm the presence of pregnancy.

#### 2.9.4. Rapid Diagnostic Tests

In the study, the RDT CareStart™ combo test (SD BIOLINE Malaria Ag *P.f/P.v* POCT test kits (Standard Diagnostic, Inc., Germany) were used and was based on lateral flow immunochromatography in cassette format, which can detect *P. falciparum* histidine-rich protein II (PfHRP-II) and other *Plasmodium* species (Pan, pLDH) (*P. vivax*, *P. malariae*, or *P. ovale*). The test was carried out on the field, and the result was read within 20 minutes, according to the manufacturer's instructions.

#### 2.9.5. Blood Film Microscopy

A thick and thin blood film was prepared and a thin smear was fixed with absolute methanol in the field and transported to the Mizan–Tepi University parasitology laboratory. It was stained using a 10% Giemsa solution for 10 minutes. The slides were examined using the oil immersion objective of the microscope by two experienced lab technicians independently. Any discordant results were resolved by a third technician, who was blind to the earlier results. The blood film was recorded as positive or negative after examining 200 high power fields of the thick smear.

#### 2.9.6. Study Variables

- Dependent Variable: asymptomatic *Plasmodium* infection
- Independent Variables: age, sex, educational status, presence of eave, presence of a visible hole in the wall, LLIN usage, LLIN coverage, animals in the house, and family size

### 2.10. Operational Definition

#### 2.10.1. Asymptomatic Malaria

It can be defined as “*Plasmodium* infections that do not lead to clinical symptoms and therefore remain undetected by fever-based surveillance systems” [17].

#### 2.10.2. Household

All individuals living in the same house as family members.

#### 2.10.3. “Sufficient”

LLIN coverage is said to be “sufficient” when the ratio of the total LLIN owned by a household to the family members is at least 0.5 (if one LLIN covers two individuals), and “not sufficient” when the ratio is less than 0.5 [18].

### 2.11. Data Quality Control

By discussing the tools before data collection, the data were checked for completeness and consistency. Clean and grease-free slides were used for blood film preparation to avoid scratches on the slides. To reduce the effect of tissue fluids, the first drop of blood was swiped away. All CareStart malaria test kits were labeled with participant identification numbers specifically given for this purpose, and the test was carried out according to the manufacturer's instructions. In all cases, the RDTs test results were determined before the microscopic results, with the microscopic examination of the thick and thin blood smears strictly blinded. Discordant slides between the microscopic readings were reanalyzed for the third time by the most experienced laboratory technician from the MTU laboratory. All laboratory procedures were conducted based on the standard operating procedures. The laboratory technicians who participated in the blood film examination were blinded to the RDTs results. At least 5% of the negative slides and all the positive slides were randomly selected for internal quality control to be checked by another laboratory technician.

### 2.12. Data Analyses

Data were entered into EpiData version 3.1 software (The EpiData Association, Odense, Denmark) and exported to SPSS version 25 (SPSS Inc., Chicago, IL, USA) for analysis. Descriptive statistics were utilized to summarize the demographic profile of the study participants. To determine the relationship between independent variables and the outcome/dependent variable, both bivariate and multivariable logistic regression analyses were employed. A *P* value less than 0.05 was taken as statistical significance.

## 3. Results

### 3.1. Sociodemographic Characteristics of the Study Participants

More than half of the participants were females, with seven of them pregnant, and nearly two-thirds were illiterate, with nearly one-fourth being farmers. Only one-fourth of them had traveled before the start of the survey, and more than two-thirds had at least five family members (Table 1).

### 3.2. Prevalence of Asymptomatic *Plasmodium* Infection in the Surveys

In the first survey, which involved 285 study participants, 11 of them were found to be positive for the malaria parasite. *Plasmodium falciparum* was the cause of seven of these cases. In the second survey, which was conducted during a major transmission season involving 281 study subjects, 27 study participants were found to be positive for *Plasmodium* species. In this survey, thirteen positive cases were identified to be *Plasmodium falciparum*, while eleven cases were accounted for *Plasmodium vivax*, and the remaining three cases were mixed infections. Sixty-four (11.3%) of the study participants reported that they had experienced malaria in the last year. Nearly one-third of the households that were included in the surveys had at least one long-lasting insecticidal net (LLIN), and 36.6% of them used it the previous night before the data collection. Multivariable analysis revealed that last night's LLIN usage (AOR = 2.448, 95% CI: 1.009–5.938, *P*=0.048), presence of eave (AOR = 4.144, 95% CI: 1.049-16.363, *P*=0.043), house sprayed in the last one year (AOR = 5.206, 95% CI: 2.176-12.455, *P* < 0.001), and presence of stagnant water nearby the home of the study participants (AOR = 2.758, 95% CI: 1.247–6.101, *P*=0.012) showed significant association with asymptomatic *Plasmodium* infections (Table 2).

### 3.3. Gametocyte Carriage among Study Participants

Of the total blood samples collected from the study participants, **seven** blood samples were found to be positive for the gametocyte stage of *Plasmodium falciparum*, which could play a great role in sustaining malaria transmission within the communities of the study areas, though people may develop resistance to the parasite's infection due to prolonged exposure to the disease. It is a well-established fact that people who serve as carriers or reservoirs of the gametocyte stage of *Plasmodium* species contribute to the vast transmission of malaria in communities living in low transmission settings, even though the study areas were previously known for their high malaria transmission. Since this study focuses on detecting asymptomatic *Plasmodium* infection, we were not able to calculate the parasitemia level (parasites per microliter of blood) because it was low in the investigation.

### 3.4. Prevalence of Asymptomatic *Plasmodium* Infection by Light Microscopy and Rapid Diagnostic Tests

**Figure 1:**
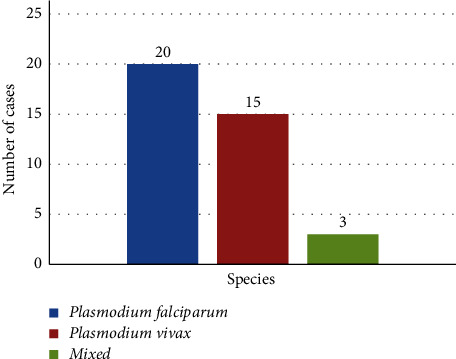
Cases of *Plasmodium* infection identified by light microscopy and rapid diagnostic tests.

A total of thirty-eight (6.7%) *Plasmodium*-positive cases were found in combined tests performed by both light microscopy and rapid diagnostic tests., of which seven (1.2%) were found to be gametocyte stages of *Plasmodium falciparum*, while three more malaria cases (all of which were *Plasmodium vivax*) were found upon screening by RDTs but not by LM (Figure 1).

## 4. Discussion

This study was conducted in districts of the Kaffa zone from April to May and September to October 2021, the two seasons which are known to favor malaria transmission across the country. Five hundred sixty-six study participants were involved in the study with all of them living in houses made of iron sheets, of which more than half were sprayed with insecticide chemicals in the last year before the survey. A high number of the study participants reported that they share a common house with their domestic animals, and nearly two-thirds of them were illiterate. Regarding LLIN coverage, nearly one-third of the study participants reported that they have sufficient bed net coverage in their home, and the level of its usage was also below half. A low prevalence of asymptomatic *Plasmodium* infection was observed in the first survey (eleven cases), whereas higher number of cases (twenty-seven) were recorded in the second survey, which was conducted during the high malaria transmission season in the study areas. Malaria prevalence was higher among female participants and those whose ages are above 15 years. It is revealed that the prevalence of asymptomatic *Plasmodium* infection found in the study was low. However, the prevalence and burden of the disease could even worsen given that ongoing malaria elimination efforts were not in place in the country, as those cases could be reservoirs of infection for the communities of the study areas. It should also be noted that the *Plasmodium* species were detected using microscopy and RDTs, both of which are less sensitive than molecular methods [19]. Therefore, the prevalence of asymptomatic *Plasmodium* infection could be higher than the recorded one in the study area. A study carried out in Jimma town also reported a low prevalence of asymptomatic malaria infection [20]. In the last decade, malaria control activities based on the distribution of LLINs, annual application of IRS, and case detection and treatment have been intensified in the Kaffa zone, as in most parts of the country.

The low asymptomatic *Plasmodium* infection prevalence discovered in this study by microscopy and RDTs compared to other similar studies could be due to factors such as storage temperature, particularly during transportation from the point of manufacture or supply to the final point of use, low sensitivity at low parasite densities, as well as exposure to adverse environmental conditions during distribution [21]. When parasitemia levels are below their detection limits of 50 and 200 parasites/Liter of blood, respectively [22], both microscopy and RDTs produce negative results. In some studies, microscopy showed better performance in detecting malaria parasites than RDTs. In a study conducted in Nigeria in 2016, microscopy detected (85.7%) of malaria parasites, which is (68.8%) for RDTs [23]. Another study from Burundi showed that 70.5% and 48.4% of the study participants were positive by microscopy and RDTs, respectively [24]. Moreover, a study on the assessment of microscopy and RDTs showed a detection rate of 66.8% and 36.8%, respectively [25]. The performance of these tools also appears to vary depending on the transmission intensity [26]. But in this study, RDTs showed relatively better parasite detection capacity than light microscopy with additional three malaria cases detected. In the surveys, last night's LLIN usage, presence of eave, house sprayed in the last year, and presence of stagnant water near the home of the study participants were significantly associated with asymptomatic *Plasmodium* infection, while educational status (being literate) seems to play a protective role against malaria infection. The study revealed almost one-third of households owned at least one-bed net, and only one-third of them used the LLIN the previous night before the survey; this calls for interventions on human behavior to effectively utilize the LLIN for malaria prevention. Much higher use of LLIN (68.3%) was reported in a study from the Limmu Seka District of Jimma Zone, Ethiopia [27].

The overall prevalence of asymptomatic *Plasmodium* infection was 6.7% (38/566), of which sixteen malaria cases were detected by light microscopy (LM) and twenty-two cases were identified by rapid diagnostic tests (RDTs), which is lower compared to studies from other parts of the worlds: Ghana (116/500, 23.2% and 156/500, 31.2%) [28], Ethiopia (17/331, 5.1%, and 30/331, 9.1%) [29] by LM and RDTs respectively, while, finding by light microscopy from Kenya and Northwest Ethiopia revealed malaria prevalence of (40/308, 12.9%) [30], and (30/251, 12%) [31] respectively. The finding of this study was higher compared to studies from Zanzibar (56/1182, 4.7%) [32], Southwest Ethiopia (7/552, 1.3%, and 20/562, 3.6%) [33], and a thesis work by the author from Dedo District, Southwest Ethiopia (6/373, 1.6%) [34] by both LM and RDTs. The above variations observed among the testing methods, light microscopy and the RDTs might be related to the performance differences/detection limits (sensitivity and specificity) of the two methods. Other possible reasons for the variations could also be related to factors such as damages to the RDT kits that may occur either during transportation or distribution or testing errors among the laboratory technicians. But these variations in the overall asymptomatic *Plasmodium* infection prevalence might be due to the differences in geographical location, i.e., the climate conditions (rainfall, temperature, and humidity), which contribute a lot to vector abundance in the study areas. Improving the use of LLINs is critically required as high coverage and use has a herd effect whereby a larger proportion of individuals utilizing the LLINs could be protected from malaria infections [35].

This could have far-reaching implications for efforts to control and eliminate malaria. Therefore, it is essential to put in place effective treatment protocols that can completely remove the gametocyte stages from the bloodstream of asymptomatic carriers of the malarial parasite, which is mainly taken by mosquitoes and responsible for the sustained transmission of the disease.

## 5. Conclusions

This study showed a low prevalence of asymptomatic *Plasmodium* infection by both diagnostic methods (light microscopy and RDTs). In both surveys, the study participants' LLINs coverage and LLINs usage were found to be low. Last night's LLIN usage, the presence of an eave, house sprayed in the last year, and presence of stagnant water nearby the home was among the associated factors that showed significant association with asymptomatic *Plasmodium* infection.

## 6. Recommendation

Although polymerase chain reaction (PCR) and loop-mediated isothermal amplification (LAMP) offers a more accurate assessment of the magnitude of asymptomatic infections; quality-assured microscopy is still officially considered the gold standard by the WHO, despite its limited performance compared to the above-advanced techniques. Moreover, surveillance that covers wide areas of the country is needed to detect all the hidden malaria cases, and on top of that mass treatment of the community (those reservoirs) in the study area is crucial.

## Figures and Tables

**Table 1:** Sociodemographic characteristics and associated factors of the study participants in selected districts of the Kaffa zone, southwest Ethiopia (*n* = 566).

Characteristics/Variables	Category	Frequency *n* (%)
Age group (year/s)	0.5–15	224 (39.6)
	>15	342 (60.4)

Sex	Male	234 (41.3)
	Female	332 (58.7)

Educational status	Literate	196 (34.6)
	Illiterate	370 (65.4)
	Farmers	128 (22.6)

Occupational status	Housewives	106 (18.7)
	Students	83 (14.7)
	Daily laborer	61 (10.8)
	Merchant	58 (10.2)
	Employed	51(9.0)
	None eligible	44 (7.8)
	Others	35 (6.2)

Presence of pregnancy	Present	7 (1.2)
	Absent	559 (98.8)

Travel history within two weeks before the survey	Yes	138 (24.4)
	No	428 (75.6)

Presence of eave	Present	439 (77.6)
	Absent	127 (22.4)

Presence of a hole in the wall	Yes	429 (75.8)
	No	137 (24.2)

Average monthly family income	<1000 ETB (22.5631 USD)	405 (71.6)
	1001–2000 ETB (22.5857-45.1263 USD)	112 (19.8)
	Above 2000 ETB (>45.1489 USD)	49 (8.7)

House sprayed with (IRS) in the last year	Yes	289 (51.1)
	No	277 (48.9)

Distance from vector breeding site	≤1 kilometer	335 (59.2)
	>1 kilometer	231 (40.8)

Malaria in the preceding year	Yes	64 (11.3)
	No	502 (88.7)

LLIN coverage	Sufficient	171 (30.2)
	Not sufficient	395 (69.8)

Last night's LLIN usage	Yes	207 (36.6)
	No	359 (63.4)

Stagnant water was found in a nearby home	Yes	224 (39.6)
	No	342 (60.4)

Animals kept in a separate house	Yes	402 (71.0)
	No	164 (29.0)

Family size	<5	165 (29.2)
	≥5	401 (0.8)

1 USD = 44.32 Ethiopian Birr (ETB) at the time of the surveys, LLIN (long-lasting insecticidal net).

**Table 2:** Bivariate and multivariable logistic regression analysis of the asymptomatic *Plasmodium* infection prevalence and associated factors in selected districts of the Kaffa zone, Southwest Ethiopia.

Variables	Category	*Plasmodium* infection		Odds ratio	
		Positive *n* (%)	Negative *n* (%)	Corollary (95% CI)	AOR (95% CI)
Age group (year/s)	0.5–15	11 (1.9)	213 (37.6)	Ref	Ref
	>15	27 (4.8	315 (55.7)	0.632 (0.293, 1.365)	1.581 (0.733, 3.414)

Sex	Male	14 (2.5)	220 (38.9)	Ref	Ref
	Female	24 (4.2)	308 (54.4)	0.788 (0.388, 1.602)	1.269 (0.624, 2.580)

Educational status	Literate	7 (1.2)	189 (33.4)	Ref	Ref
	Illiterate	31 (5.5)	339 (59.9)	2.730 (1.064, 7.007)^*∗*^	0.366 (0.143, 0.940)^*∗*^
	Farmers	11(1.9)	117 (20.7)	0.520 (0.095, 2.832)	1.923 (0.353, 10.475)

Occupational status	Housewives	6 (1.1)	100 (17.7)	0.903 (0.157, 5.175)	1.108 (0.193, 6.352)
	Students	4 (0.7)	79 (14.0)	1.353 (0.208, 8.824)	0.739 (0.113, 4.817)
	Daily laborer	2 (0.4)	59 (10.4)	1.758 (0.204, 15.146)	0.569 (0.066, 4.903)
	Merchant	3 (0.5)	55 (9.7)	1.180 (0.166, 8.391)	0.847 (0.119, 6.022)
	Employed	5 (0.9)	46 (8.1)	0.418 (0.065, 2.682)	2.392 (0.373, 15.343)
	None eligible	5 (0.9)	39 (6.9)	0.341 (0.052, 2.214)	2.934 (0.452, 19.063)
	Others	2 (0.4)	33 (5.8)	Ref	Ref

Presence pregnancy	Present	3 (0.5)	8 (1.4)	Ref	Ref
	Absent	35 (6.2)	520 (91.9)	4.998 (1.109, 22.530)^*∗*^	0.200 (0.044, 0.902)^*∗*^

Travel history 2 weeks before the survey	Yes	4 (0.7)	134 (23.7)	Ref	Ref
	No	34 (6.0)	394 (69.6)	0.415 (0.140, 1.223)	2.412 (0.818, 7.118)

Malaria in the preceding year	Yes	5 (0.9)	59 (10.4)	Ref	Ref
	No	33 (5.8)	469 (82.9)	1.456 (0.518, 4.095)	0.687 (0.244, 1.932)

Last night's usage LLIN	Yes	7 (1.2)	200 (35.3)	Ref	Ref
	No	31 (5.5)	328 (58.0)	0.408 (0.168, 0.991)^*∗*^	2.448 (1.009, 5.938)^*∗*^

LLIN coverage	Sufficient	12 (2.1)	159 (28.1)	Ref	Ref
	Not sufficient	26 (4.6)	369 (65.2)	1.164 (0.549, 2.468)	0.859 (0.405, 1.822)

Presence of eave	Present	32 (5.7)	407 (71.9)	0.241 (0.061, 0.953)^*∗*^	4.144 (1.049, 16.363)^*∗*^
	Absent	6 (1.1)	121 (21.4)	Ref	Ref

Presence of a hole in the wall	Yes	28 (4.9)	401 (70.8)	3.132 (1.027, 9.557)^*∗*^	0.319 (0.105, 0.974)^*∗*^
	No	10 (1.8)	127 (22.4)	Ref	Ref

House sprayed in the last year	Yes	7 (1.2)	282 (49.8)	Ref	Ref
	No	31 (5.5)	246 (43.5)	0.192 (0.080, 0.460)^*∗*^	5.206 (2.176, 12.455)^*∗*^

Distance from vector breeding site in meter	≤1 kilometer	21 (3.7	314 (55.5)	0.959 (0.439, 2.097)	1.042 (0.477, 2.278)
	>1 kilometer	17 (3.0)	214 (37.8)	Ref	Ref

Average monthly family income	Below 1000 ETB (22.5631 USD)	25 (4.4)	380 (67.1)	3.433 (1.032, 11.420)^*∗*^	0.291 (0.088, 0.969)^*∗*^
	1001−2000ETB (22.5857-45.1263 USD)	8 (1.4)	104 (18.4)	2.140 (0.522, 8.779)	0.467 (0.114, 1.917)
	Above 2000 ETB (>45.1489 USD)	5 (0.9)	44 (7.8)	Ref	Ref

Animals kept in a separate house	Yes	23 (4.1)	379 (67.0)	1.604 (0.743, 3.464)	0.623 (0.289, 1.346)
	No	15 (2.7)	149 (26.3)	Ref	Ref

Family size (number)	<5	11 (1.9)	154 (27.2)	Ref	Ref
	≥5	27 (4.8)	374 (66.1)	0.691 (0.317, 1.508)	1.447 (0.663, 3.159)

^*∗*^Significant at *P* < 0.05, Ref = represents reference group, USD refers to the US dollar, ETB stands for Ethiopian birr.

## Data Availability

The data related to this research can be obtained from the corresponding author upon reasonable request.

## Abbreviations/Acronyms

IRS: Indoor residual spraying
LAMP: Loop-mediated isothermal amplification
LLIN: Long-lasting insecticidal net
LM: Light microscopy
ELISA: Enzyme-linked immunosorbent assay
PCR: Polymerase chain reaction
RBC: Red blood cell
RDT: Rapid diagnostic test
WHO: World Health Organization.
